# The Impact of Demographic and Economic Change on the Australian Generational Economy: Financial Sustainability, Intergenerational Inequality, and Material Living Standards

**DOI:** 10.3389/fpubh.2022.798298

**Published:** 2022-06-14

**Authors:** James Mahmud Rice, Tom Wilson, Jeromey B. Temple, Peter McDonald

**Affiliations:** Demography and Ageing Unit, Melbourne School of Population and Global Health, University of Melbourne, Melbourne, VIC, Australia

**Keywords:** Australia, consumption, fertility, income, inequality, intergenerational, migration, mortality

## Abstract

The generational economy—which is that aspect of the economy that pertains to the economic activities of, and the economic relationships between, different ages and generations—can be evaluated on the basis of a number of different criteria. The most critical of these include the financial sustainability of the generational economy, the intergenerational inequality that the generational economy creates, and the material living standards associated with the generational economy. How the generational economy performs in terms of these three criteria is, moreover, shaped by underlying processes of demographic and economic change. This paper examines how the Australian generational economy can be expected to perform in coming decades in terms of financial sustainability, intergenerational inequality, and material living standards. How the performance of the Australian generational economy is shaped by variations in fertility, mortality, overseas migration, and labour-income growth is also assessed. The results reported in the paper indicate that, because of population aging, consumption can only grow at a substantially lower rate than labour income if financial sustainability is to be maintained. These results also suggest that increasing overseas migration is a distinctly useful policy tool for meeting the challenges posed by population aging, since increasing overseas migration both increases material living standards and decreases intergenerational inequality.

## Introduction

Like most high-income countries, Australia has experienced substantial demographic changes over an extended time period which have led to the aging of its population. Under the influence of declining fertility and mortality rates, countered to an extent by overseas migration, between 1980 and 2020 the share of Australia's population that is 65 years of age or older has risen from 9.6 to 16.3%. Over the same time period, the share of the population that is 19 years of age or younger has fallen from 34.2 to 24.4%, while the share of the population between 20 and 64 has risen from 56.2 to 59.3% (1).

A clear patterning by age exists with respect to economic life. Workforce participation and labor income are low during younger and older ages and high during the middle of the life cycle, while consumption is more evenly distributed. Consequently, deficits exist between labor income and consumption during younger and older ages that are in part funded by surpluses of labor income over consumption during the middle of the life cycle (2–5). Because of this patterning of economic life by age, population aging leads to a decline in the number of workers in a country relative to the number of consumers, or more precisely to a decline in the income a country earns through its own labor relative to its consumption. This increasing disjuncture between labor income and consumption places growing pressure on a country's future material standards of living.

That aspect of the economy that pertains to the economic activities of—as well as the economic relationships between—different ages and generations is referred to here as the “generational economy” (6). Through its impact on the disjuncture between labor income and consumption, population aging places the financial sustainability of the generational economy at risk.

Research into the impact of population aging on the financial sustainability of the generational economy is a task taken up by researchers around the world. In Australia in recent years this research has included work undertaken by researchers at the ARC Centre of Excellence in Population Aging Research (3, 7, 8), Australian federal and state governments (9–12), and others (13, 14).

Financial sustainability is one criterion on the basis of which the performance of generational economies can be evaluated. It is not, however, the only such criterion. How generational economies perform can also be evaluated on the basis of the intergenerational inequality, or the inequality between birth cohorts, that they create (13, 15–19). Although they are often conflated, financial sustainability and intergenerational inequality are distinct concepts (13, 16, 18, 19). An economy that is financially sustainable, for example, can manifest a large or a small level of inequality between birth cohorts. Similarly, an economy that manifests a high level of inequality between birth cohorts can be financially sustainable or unsustainable.

This paper has two principal ambitions. Firstly, it aims to examine how the Australian generational economy might perform in coming decades in terms of both intergenerational inequality and financial sustainability, as well as material living standards. Secondly, it aims to assess how demographic and economic changes might affect the performance of the Australian generational economy vis-a-vis these three evaluative criteria. To this end, the performance of the Australian generational economy is assessed under a variety of demographic and economic scenarios. Demographic scenarios are delineated on the basis of projected changes in the demographic processes of fertility, mortality, and overseas migration. Economic scenarios are delineated on the basis of projected changes in labor-income growth.

The following section describes the materials and methods on which this paper is based, including how financial sustainability, intergenerational inequality, and material living standards are assessed in the paper and the detail of how the demographic and economic scenarios are constructed. Results are then presented, followed by a discussion of these results and a conclusion.

## Materials and Methods

### Evaluating the Generational Economy

This section describes how the performance of a generational economy is evaluated in this paper. Three criteria are discussed: the financial sustainability of a generational economy, the intergenerational inequality a generational economy creates, and the material living standards associated with a generational economy.

The focus here is on the performance of an economy in a broad sense, rather than on the performance of more narrow components of an economy. The focus is on the financial sustainability, intergenerational inequality, and material living standards associated with an economy more broadly, for example, rather than on the financial sustainability, intergenerational inequality, and material living standards associated with particular government budgets or particular pension programs. Findings about more narrow components of an economy have been shown to not hold when the focus is widened to the economy more broadly, and vice versa (18–20).

#### Financial Sustainability

In this paper the financial sustainability of a generational economy is assessed by way of a summary measure based on the support ratio. The support ratio (*SR*) for year *t* can be defined as follows (20–23):


$$\begin{array}{l} {SR}_{t}=\frac{\sum_{a=0}^{a_{oldest}}L_{at}N_{at}}{\sum_{a=0}^{a_{oldest}}C_{at}N_{at}} \end{array}$$

where: *t* = year; *a* = age; *a*_*oldest*_ = the oldest age in the population; *L*_*at*_ = per capita labor income at age *a* in year *t*; *C*_*at*_ = per capita consumption at age *a* in year *t*; and *N*_*at*_ = number of people at age *a* in year *t*. The support ratio as defined here has also been referred to as the economic support ratio (18, 19).

This ratio compares the amount of labor income in an economy in year *t* to the amount of consumption. A summary measure that compares labor income and consumption across an extended time period—labeled here as the time-interval support ratio (*TISR*)—can be defined in the following manner:


$$\begin{array}{l} {TISR}_{t_{start},t_{end}}=\frac{\sum_{t=t_{start}}^{t_{end}}\sum_{a=0}^{a_{oldest}}L_{at}N_{at}{\left(1\;+\;R\right)}^{-\left(t-t_{start}\right)}}{\sum_{t=t_{start}}^{t_{end}}\sum_{a=0}^{a_{oldest}}C_{at}N_{at}{\left(1\;+\;R\right)}^{-\left(t-t_{start}\right)}} \end{array}$$

where: *t*_*start*_ = the year in which the time period starts; *t*_*end*_ = the year in which the time period ends; and *R* = the annual real discount rate.

The time-interval support ratio is equal to the total present value of labor income during the time period between *t*_*start*_ and *t*_*end*_, expressed as a proportion of the total present value of consumption during this time period. It indicates the proportion of consumption that can be funded by labor income. The remaining consumption must be funded by resources other than labor income. These other resources can include those based on the utilization of assets, such as asset income and dissaving, as well as transfers received from other economies.

The higher the value of the time-interval support ratio, the higher the proportion of consumption that can be funded by labor income and the lower the proportion of consumption that must be funded by resources such as asset income, dissaving, and transfers received from other economies. Here higher values of the time-interval support ratio are taken to indicate higher levels of financial sustainability.

If an economy maintains the same support ratio in each year during the period between *t*_*start*_ and *t*_*end*_, the time-interval support ratio for this period will simply equal the support ratio.

In this paper the real discount rate is set to 3% per annum. This discount rate is set to the risk-free rate of return, following Lee, McCarthy, Sefton, and Sambt (24). The weighted average risk-free rate of return in Australia is estimated by Harrison to be 3% per annum (25).

#### Intergenerational Inequality

The approach to intergenerational inequality, or inequality between birth cohorts, in this paper builds on earlier work by Rice, Temple, and McDonald (4, 26). Birth cohorts are, of course, defined by year of birth. The particular year in which a person happens to be born is an accident of birth, much like the way the particular gender or ethnicity a person happens to be born is an accident of birth. In this paper inequality between birth cohorts is assessed in a way that is similar to how inequality between genders or ethnicities is commonly assessed.

Here inequality between birth cohorts is evaluated on the basis of inequalities in the material living standards that different birth cohorts experience over their lifetimes. Theories of fairness that focus on equality of outcomes would argue that inequalities between birth cohorts in terms of material living standards are intrinsically unfair. Theories of fairness that focus on equality of opportunities could argue that inequalities between birth cohorts in terms of material living standards are unfair because, to a large extent, these inequalities reflect the unequal opportunities that are open to different birth cohorts. Cohorts who happen to be born during times of low material living standards, for example, have fewer opportunities to enjoy high material living standards when compared to cohorts who happen to be born during times of high material living standards (26).

Transfers and redistribution between birth cohorts are similarly evaluated on the basis of the effect that they have on inequalities in the material living standards that different birth cohorts experience over their lifetimes. If some cohorts experience lower material living standards over their lifetimes than others, transfers and redistribution between cohorts could decrease inequality between cohorts. Moreover, where there are inequalities in the material living standards that different cohorts experience over their lifetimes, equality between cohorts does not require that the transfers each cohort receives from other cohorts over its lifetime be balanced by the transfers it pays to other cohorts. In other words, there is no requirement that there be no net transfers or redistribution between cohorts over cohorts' lifetimes. This is similar to how transfers and redistribution between genders or ethnicities are commonly assessed. Where there are inequalities in the material living standards that different genders or ethnicities experience over their lifetimes, for example, gender equality or ethnic equality does not require that there be no net transfers or redistribution between genders or ethnicities.

The approach to intergenerational inequality in this paper does differ from other approaches that assess inequality between birth cohorts on the basis of inequalities in the net transfers between birth cohorts. One example of these other approaches is the notion that intergenerational equality is attained when birth cohorts “pay their way” without subsidizing or being subsidized by other birth cohorts, even if this involves some birth cohorts experiencing lower material living standards than others (13, 17).

In this paper material living standards are measured by levels of consumption and inequality between birth cohorts is evaluated on the basis of inequalities in the consumption that different birth cohorts experience over their lifetimes. In more specific terms, inequality between birth cohorts is assessed by way of two summary measures of inequality in the consumption experienced by different birth cohorts. These two measures are described in the following sections.

##### The IGI Index for Consumption

The first of these measures is the IGI index, applied in this case to consumption. The IGI index is an indicator of intergenerational inequality that is described in detail in a recent article by Rice, Temple, and McDonald (26). At its core the IGI index summarizes inequality between birth cohorts in, say, consumption by calculating the Gini coefficient across estimates of the relative inequalities between birth cohorts in lifetime consumption. When estimated for the time period between *t*_*start*_ and *t*_*end*_, the IGI index is calculated as follows:


$$\begin{array}{l} {IGI}_{t_{start},t_{end}}=\frac{\sum_{b=earliest}^{b=latest}\sum_{d=earliest}^{d=latest}\left(W_{b}W_{d}|C_{b}^{*}\;-\;C_{d}^{*}|\right)}{2{\left(\sum_{b=earliest}^{b=latest}W_{b}\right)}^{2}\mu } \end{array}$$

where: *b* (and *d*) = the years of birth that define the birth cohorts for which data is available between *t*_*start*_ and *t*_*end*_; *W*_*b*_ = weight for the birth cohort born in year *b*; $C_{b}^{*}$ = the lifetime consumption of the birth cohort born in year *b* relative to that of other birth cohorts, estimated as described in the recent article by Rice, Temple, and McDonald; and μ = mean of C^*^ across all birth cohorts. In this paper the weights allocated to birth cohorts reflect population distributions by birth cohort. These population distributions in turn reflect the demographic processes of fertility, mortality, and overseas migration.

Like the Gini coefficient, the IGI index for consumption satisfies the Pigou–Dalton condition (or the principle of transfers), which is to say that whenever consumption is transferred or redistributed from one birth cohort to another birth cohort with a lower consumption, this is registered by the IGI index for consumption as a decrease in inequality, irrespective of the position of these two cohorts within the broader distribution of consumption. Like the Gini coefficient, the IGI index for consumption has a lower bound equal to 0 and an upper bound that approaches 1 (when the number of birth cohorts is large). The lower bound is attained in the extreme case of absolute equality, in which all birth cohorts experience the same lifetime consumption. The upper bound in attained at the other extreme in which all consumption is experienced by just one birth cohort (26).

##### Redistributing Consumption

The second summary measure of inequality in the consumption experienced by different birth cohorts begins with the total present value of consumption during an extended time period (which is the denominator for the time-interval support ratio described above). This measure then assesses what proportion of this total present value must be redistributed in order to attain absolute intergenerational equality in consumption during this time period. This measure is referred to here as the “indicator of equalizing redistribution for consumption”.

The analysis presented in this paper focuses on 101 single years of age from 0 to 100 during the 101 years from 2020 to 2120, inclusive. The outcome of this is that there is only one birth cohort—that born in 2020—for which a completed life cycle is available. With this in mind, this paper envisages absolute intergenerational equality in consumption between 2020 and 2120 as the state in which all birth cohorts experience the same, reference profile of per capita consumption by age, with this reference profile being based on the completed profile of per capita consumption by age of the 2020 birth cohort—subject to the condition that the total present value of consumption during the time period between 2020 and 2120 does not change.

Within the context of these particular ages and years, the total present value of consumption (*Total PVC*) between 2020 and 2120 can be defined in the following way:


$$\begin{array}{l} Total\;{PVC}_{t_{start}=2020,t_{end}=2120}\;=\;\overset{2120}{\underset{t=2020}{\sum }}\overset{100}{\underset{a=0}{\sum }}C_{at}N_{at}{\left(1\;+\;R\right)}^{-\left(t-2020\right)} \end{array}$$

Per capita consumption at age *a* for the 2020 birth cohort can be labeled *C2020*_*a*_ (specifying a year *t* is unnecessary, since for the 2020 birth cohort *t* will always equal *a* plus 2020). If all birth cohorts experience a reference profile of per capita consumption by age that is equal to that of the 2020 birth cohort (that is, *C2020*_*a*_), the resulting total present value of consumption (*Total PVC2020*) between 2020 and 2120 would be:


$$\begin{array}{lll} Total\;{PVC2020}_{t_{start}=2020,t_{end}=2120} & = & \overset{2120}{\underset{t=2020}{\sum }}\overset{100}{\underset{a=0}{\sum }}{C2020}_{a}N_{at}{\left(1+R\right)}^{-\left(t-2020\right)} \end{array}$$

These two totals are unlikely to be equal. Because of this, per capita consumption at age *a* in the reference per capita age profile for consumption (*CREF*_*a*_) is not set to *C2020*_*a*_, but rather to an adjusted version of *C2020*_*a*_, as follows:


$$\begin{array}{l} {CREF}_{a}\;=\;{C2020}_{a}\times \left(\frac{{Total\;PVC}_{t_{start}=2020,t_{end}=2120}}{{Total\;PVC2020}_{t_{start}=2020,t_{end}=2120}}\right) \end{array}$$

In order for all birth cohorts to experience this reference profile of per capita consumption by age, consumption-related redistributive transfers must be received by some individuals and paid by others. The per capita consumption-related redistributive transfers (τ) received by individuals at age *a* in year *t* are calculated as follows:


$$\begin{array}{l} \tau_{at}\;=\;{CREF}_{a}\;-\;C_{at} \end{array}$$

Positive values of τ indicate that redistributive transfers are received, while negative values indicate that transfers are paid. (The total present value of redistributive transfers between 2020 and 2120 will equal zero, with the total present value of transfers received being perfectly matched by the total present value of transfers paid.)

The indicator of equalizing redistribution for consumption (*ER*) for the time period between 2020 and 2120 is then calculated in the following manner:


$$\begin{array}{l} {ER}_{t_{start}=2020,t_{end}=2120}\;=\;\frac{\sum_{t=2020}^{2120}\sum_{a=0}^{100}|\tau_{at}|\;{N_{at}\;(1\;+\;R)}^{-\left(t-2020\right)}}{2\;\times \;{Total\;PVC}_{t_{start}=2020,t_{end}=2120}} \end{array}$$

This indicator measures the proportion of consumption during an extended time period that must be redistributed in order to attain absolute intergenerational equality in consumption during this time period. It varies between 0 and 1, with higher values indicating higher levels of intergenerational inequality.

Some might argue that redistributing consumption between birth cohorts during a particular, extended time period is questionable in terms of intergenerational inequality over a longer, more extensive time period. After all, increasing consumption for low-consumption cohorts within a particular period increases the inequality between these cohorts and cohorts outside that particular period who experience even less consumption. Similarly, decreasing consumption of within-period, high-consumption cohorts increases the inequality between these cohorts and outside-period cohorts who experience even higher consumption. An argument of this kind, however, runs directly counter to the Pigou–Dalton condition. This condition suggests that redistributing consumption from one cohort to another cohort with a lower consumption during a particular time period does indeed decrease inequality, irrespective of whether cohorts other than these two experience more or less consumption.

The IGI index for consumption and the indicator of equalizing redistribution for consumption embody different concepts related to intergenerational inequality and are calculated in different ways. Consequently, they will not always yield the same results. Of the two, the IGI index is the more sound measure of intergenerational inequality. This assessment is supported by the Pigou–Dalton condition. While the IGI index satisfies the Pigou–Dalton condition, the indicator of equalizing redistribution does not. The latter, though, does provide a useful supplement to the former. While the IGI index measures intergenerational inequality, the indicator of equalizing redistribution emphasizes how this inequality might be addressed through redistribution.

#### Material Living Standards

As mentioned above, material living standards are measured by levels of consumption. Turning specifically to change over time in material living standards, in this paper increases in material living standards between *t*_*start*_ and *t*_*end*_ are assessed by way of the growth rates of age-specific, per capita consumption (that is, *C*_*at*_) between *t*_*start*_ and *t*_*end*_.

### Demographic Scenarios

The demographic scenarios in this paper are based on population projections for Australia from 2020 to 2120 produced with a standard cohort-component population projection model (27). Three possible future trajectories for each of the demographic processes of fertility, mortality, and overseas migration were formulated (low, medium, and high), resulting in a total of 27 projections. Medium assumptions for fertility and overseas migration reflect levels observed over the last few years, while for mortality they continue long-term trends recorded over the last five decades.

Fertility assumptions were trended in over the first few years of the projection horizon from recently observed fertility rates and then held constant at the chosen long-term levels. These long-term assumptions for the total fertility rate were 1.50 (low), 1.65 (medium), and 1.80 (high). In all of the fertility scenarios, a gradual aging of the fertility age profile was assumed based on projections obtained from the parameterised Peristera and Kostaki fertility age profile model (28).

Mortality was assumed to continue its gradual, long-term historical decline and was projected using a modified version of Ediev's extrapolative model (29). No adjustments in mortality assumptions were made due to COVID-19, since to date no substantial effect on mortality has been observed in Australia. Low, medium, and high assumptions were prepared in terms of life expectancy at birth, with gradual increases in, and divergence of, the three life expectancy trajectories assumed over time. By the end of the projection horizon in 2120, life expectancy was assumed to have risen to 91.7 years for females and 90.9 years for males (low), 95.7 years for females and 94.9 for males (medium), or 99.7 years for females and 98.9 for males (high).

Overseas migration assumptions were summarized in terms of annual total net overseas migration gains (although the projection model uses separate age and sex immigration and emigration flows in its calculations). All three overseas migration assumptions were trended in over the first few years of the projection horizon from recent negative net migration values resulting from the closure of the Australian border due to the COVID-19 pandemic. The net overseas migration assumptions were then assumed to remain fixed, with the low scenario set at 150,000 per annum, the medium scenario at 210,000, and the high scenario at 270,000.

While a total of 27 population projections were produced, the demographic scenarios discussed in this paper are based on only seven of these. A “Medium” demographic scenario is defined by medium assumptions for fertility, mortality, and overseas migration and constitutes a baseline against which the other scenarios are compared. In order to investigate the impacts of fertility, mortality, and overseas migration on financial sustainability, intergenerational inequality, and material living standards, the other scenarios share the same medium assumptions as the Medium demographic scenario, apart from differing from that scenario in terms of assumptions for, in turn, fertility, mortality, and overseas migration. “Low Fertility” and “High Fertility” scenarios are defined by low and high assumptions for fertility, respectively. “Low Mortality” and “High Mortality” scenarios are defined by low and high assumptions for mortality. “Low Migration” and “High Migration” scenarios are defined by low and high assumptions for overseas migration.

### Economic Scenarios

The economic scenarios in this paper begin with estimates of mean or per capita amounts of labor income and consumption among individuals, broken down by single year of age, sourced from the Australian National Transfer Accounts (NTA). The Australian NTA is a system of macroeconomic accounts that measures current economic flows by age in a manner consistent with the Australian System of National Accounts. A wide range of data sources and methods have been marshaled in order to construct these accounts. Of the data sources used, the most crucial have been the surveys of household expenditure, income, and housing that have been conducted by the Australian Bureau of Statistics, as well as the Australian System of National Accounts. Further detail about the Australian NTA can be found in publications by Rice, Temple, and McDonald (3, 5).

The estimates of labor income and consumption contained within the Australian NTA are unusually comprehensive. They incorporate people who live in private dwellings as well as those who live in residential aged care. The estimates of labor income include wages and salaries paid in cash, fringe benefits received in kind, and the share of self-employment income that can be considered to be a return to labor. The estimates of consumption include individual as well as collective consumption. These estimates include consumption funded by the public sector, as well as that funded by the private sector.

Australian NTA estimates of labor income and consumption are currently available for six financial years during the 28-year time period between 1981–82 and 2009–10. For the purposes of this paper, estimates of per capita labor income and consumption by age for 2020 were derived by inflating Australian NTA estimates for 2009–10 so that, when multiplied by the population age distribution for 2020, these estimates align exactly with macroeconomic benchmarks for labor income and consumption during 2020. These macroeconomic benchmarks for 2020 were derived from the Australian System of National Accounts, together with other information sources.

The economic scenarios are defined by the rates at which age-specific, per capita labor income is assumed to grow, in real terms, during the period between 2020 and 2120.

A baseline, “Medium” economic scenario assumes that age-specific, per capita labor income grows at a constant real rate of 1.5% per annum for all age groups. This growth rate aligns with the baseline growth rate for labor productivity used in the Australian government's 2021 Intergenerational Report. This report assumes that in the long-term productivity grows by 1.5% per annum, which is equal to the average growth rate in productivity over the 30 years leading up to 2018–19. Real wages are assumed in this report to increase in line with growth in productivity (9). Estimates based on the Australian NTA likewise indicate that age-standardized, per capita labor income grew by 1.5% per annum in real terms over the course of the 28-year time period between 1981–82 and 2009–10.

Lower and higher economic scenarios are delineated through varying the per capita labor-income growth rate around this 1.5% baseline, from 1.2 to 1.8% per annum. The 1.2% per annum growth rate mirrors recent low labor-productivity levels and aligns with the lower productivity growth rate used in the 2021 Intergenerational Report and the Australian government's Beyond the Budget 2021–22 report (9–11). The 1.8% per annum growth rate mirrors the productivity boom of the early 1990s and aligns with the higher productivity growth rate used in the Beyond the Budget 2021–22 report (10, 11).

In each economic scenario, consumption is assumed to adjust to demographic and labor-income changes in such a way that the financial sustainability of the generational economy is maintained at a certain level. More specifically, between 2020 and 2120 age-specific, per capita consumption is assumed to grow at a constant real rate for all age groups such that the time-interval support ratio is maintained at a level equal to the support ratio for 2020.

## Results

### The Medium Scenario

The analytical approach adopted in this paper is to estimate an overall, “Medium” scenario for the Australian generational economy—in which medium assumptions are used for fertility, mortality, overseas migration, and labor-income growth—and then to examine how altering these assumptions shapes material living standards and intergenerational inequality while financial sustainability is maintained at a certain level. In this section an account of this Medium scenario will be provided.

**Figure 1:**
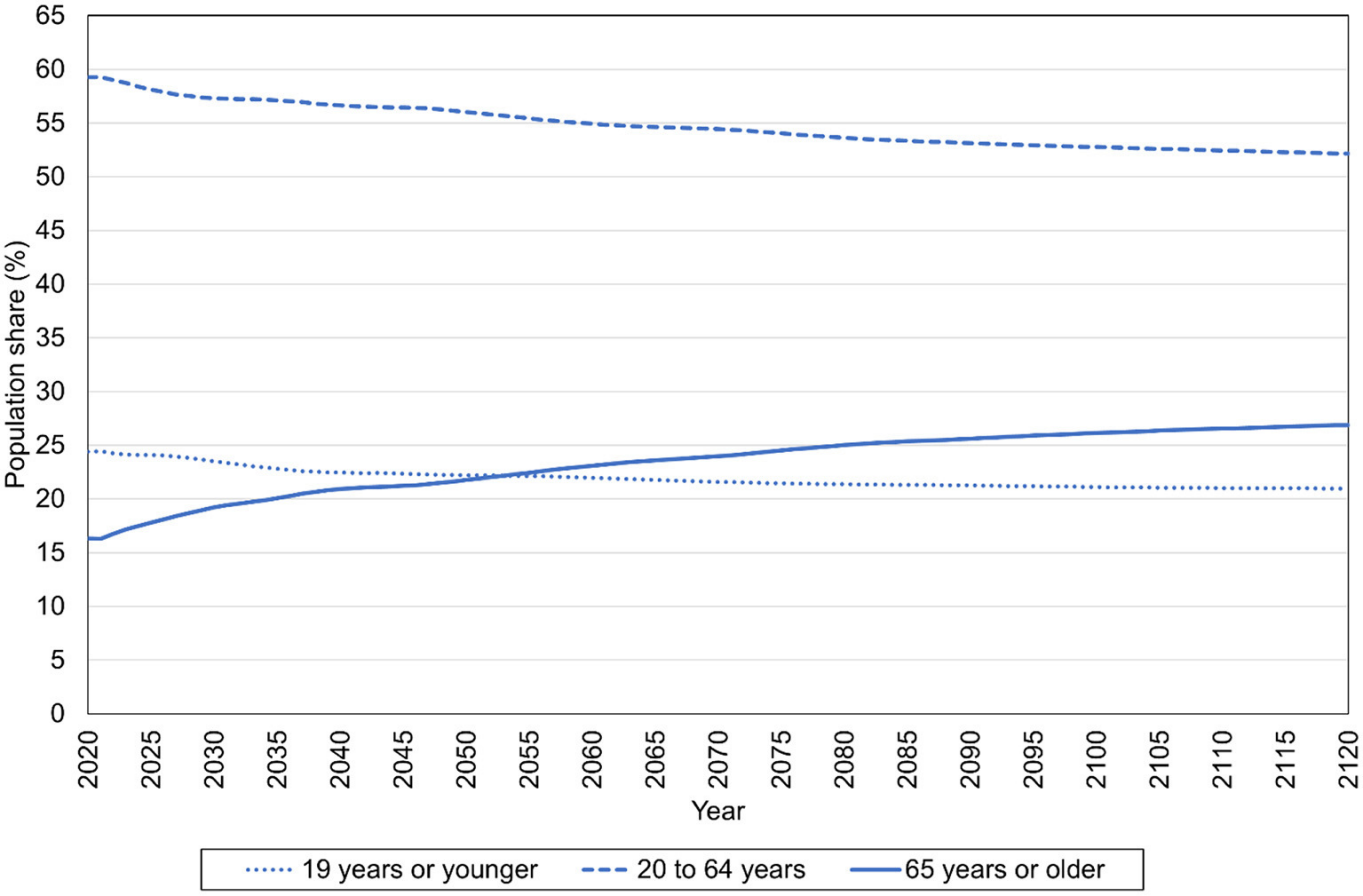
Population shares of different age groups, 2020–2120, Medium scenario, Australia (%).

Figure 1 describes the shares of Australia's population that are 19 years of age or younger, between 20 and 64 years of age, and 65 years of age or older between 2020 and 2120 in the Medium scenario. Population aging is evident in the Medium scenario, with the share of the population that is 65 years of age or older rising from 16.3% in 2020 to 24.0% in 2070, and then rising more to 26.9% in 2120. The share of the population that is 19 years of age or younger falls from 24.4% in 2020 to 21.6% in 2070, and then falls further to 21.0% in 2120. The share of the population between 20 and 64 falls as well, from 59.3% in 2020 to 54.4% in 2070 and then to 52.1% in 2120. The population share of people 65 or older surpasses that of people 19 or younger in 2053.

In the Medium scenario age-specific, per capita labor income grows at a rate of 1.5% per annum between 2020 and 2120. Because of the population aging described in Figure 1, age-specific, per capita consumption can only grow at a substantially lower rate—namely, 1.27% per annum—if the time-interval support ratio is to be maintained at a level equal to the support ratio for 2020 (This support ratio is equal to 0.787).

This consumption growth rate means that people born in one year will enjoy levels of consumption at each age that are 1.27% higher than those experienced by people who happen to have been born one year earlier. This inequality between birth cohorts, when combined with the distribution of the population by birth cohort in the Medium scenario, leads to a value for the IGI index for consumption that is equal to 0.311. The indicator of equalizing redistribution for consumption is equal to 0.179, which suggests that 17.9% of the total present value of consumption between 2020 and 2120 would need to be redistributed in order to address this inequality between birth cohorts and for equality between birth cohorts to be attained. Since cohorts born in later years will enjoy higher levels of consumption than those experienced by cohorts born in earlier years, this redistribution of consumption will involve redistribution from later to earlier cohorts.

The following sections will examine how altering fertility, mortality, overseas migration, and labor-income growth might alter material living standards and intergenerational inequality, assuming that financial sustainability is maintained at a certain level.

### Labor-Income Growth

**Figure 2:**
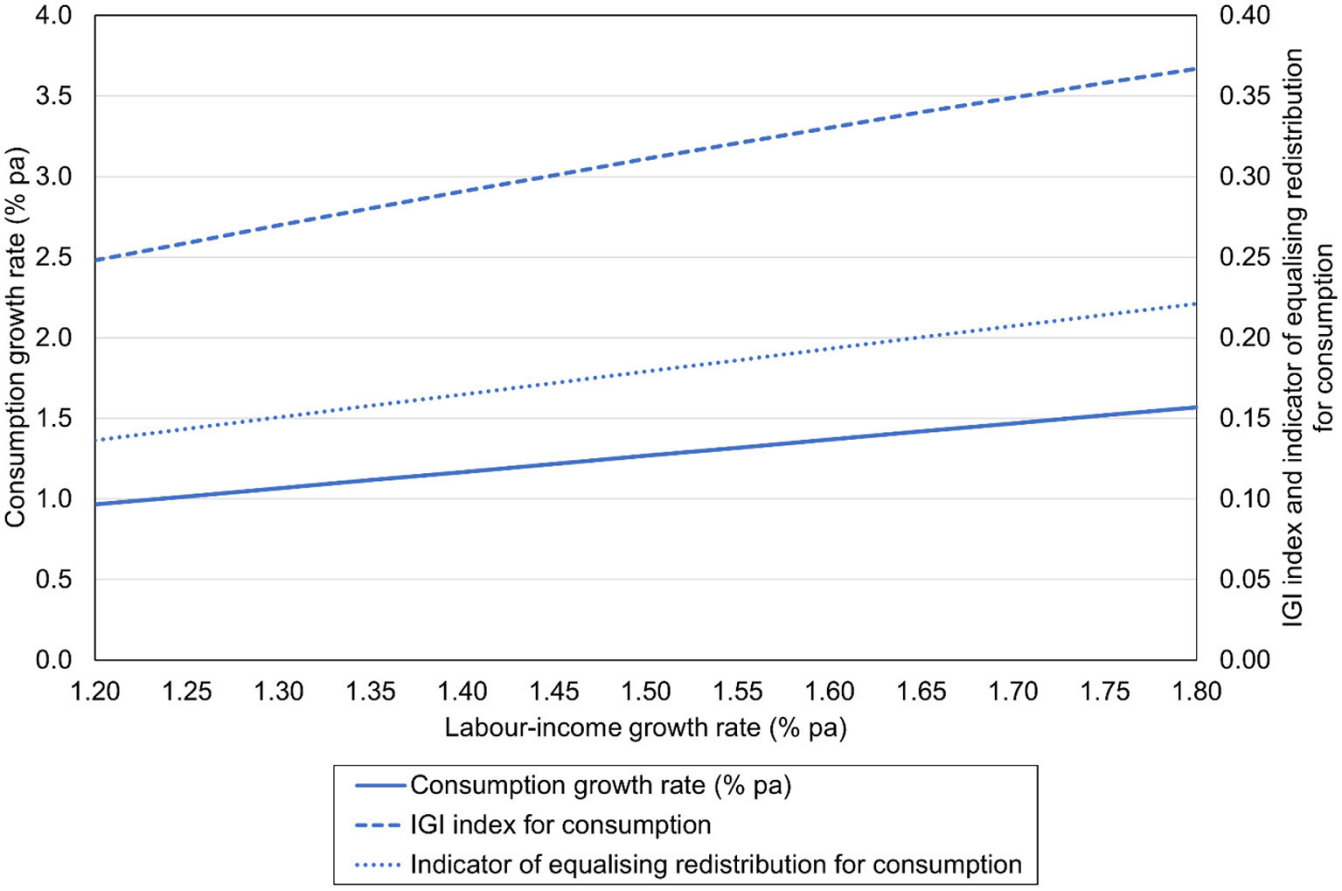
Age-specific, per capita consumption growth rates, the IGI index for consumption, and the indicator of equalizing redistribution for consumption by age-specific, per capita labor-income growth rates, 2020–2120, Australia. This figure assumes that the Medium demographic scenario holds.

Figure 2 describes how altering the rate of growth of age-specific, per capita labor income shapes the rate at which age-specific, per capita consumption grows, assuming that the time-interval support ratio is maintained at a level equal to the support ratio for 2020. This figure also depicts how the IGI index and the indicator of equalizing redistribution for consumption vary with the labor-income growth rate, through the latter's influence on the consumption growth rate. This figure assumes that the Medium demographic scenario holds in Australia between 2020 and 2120.

Increasing the labor-income growth rate allows the consumption growth rate to be higher. A labor-income growth rate of 1.5% per annum allows a consumption growth rate of 1.27% per annum, as mentioned in the previous section. Labor-income growth rates of 1.2 and 1.8% per annum are associated with consumption growth rates of 0.97 and 1.57%, respectively. Generally speaking, the consumption growth rate must be 0.23 percentage points below the labor-income growth rate if the time-interval support ratio is to be maintained at a level equal to the support ratio for 2020.

Because of this link between the labor-income and consumption growth rates, increases in the labor-income growth rate are associated with rises in the IGI index and the indicator of equalizing redistribution for consumption. Increasing the labor-income growth rate from 1.2 to 1.5 to 1.8% per annum leads the IGI index to rise from 0.248 to 0.311 to 0.367. Increasing the labor-income growth rate in this way leads the indicator of equalizing redistribution to rise from 0.136 to 0.179 to 0.221.

In this way increasing the labor-income growth rate has the effect of raising material living standards, but it also has a second effect, namely, that of increasing intergenerational inequality.

### Fertility

Table 1 describes the impacts that fertility, mortality, and overseas migration have on material living standards and intergenerational inequality, assuming that financial sustainability is maintained at a certain level. More specifically, this table presents, for each of the seven demographic scenarios described above, values for the age-specific, per capita consumption growth rate, the IGI index for consumption, and the indicator of equalizing redistribution for consumption, assuming that the Medium economic scenario holds. The population shares of different age groups in 2120 are also presented.

**Table 1:** Population shares of different age groups, age-specific, per capita consumption growth rates, the IGI index for consumption, and the indicator of equalizing redistribution for consumption under different demographic scenarios, 2020–2120, Australia.

**Demographic scenario**	**Assumptions**			**Population shares of age groups, 2120 (%)**			**Consumption growth rate (% pa)**	**IGI index for consumption**	**Indicator of equalizing redistribution for consumption**
	**Fertility**	**Mortality**	**Overseas migration**	**≤19 years**	**20–64 years**	**≥65 years**			
Medium	Medium	Medium	Medium	21.0	52.1	26.9	1.27	0.311	0.179
Low fertility	Low	Medium	Medium	19.8	52.4	27.8	1.27	0.319	0.177
High fertility	High	Medium	Medium	22.2	51.9	25.9	1.26	0.303	0.181
Low mortality	Medium	Low	Medium	20.3	50.7	29.0	1.22	0.299	0.172
High mortality	Medium	High	Medium	21.6	53.5	24.9	1.31	0.323	0.185
Low migration	Medium	Medium	Low	20.4	51.4	28.3	1.22	0.316	0.170
High migration	Medium	Medium	High	21.3	52.6	26.1	1.30	0.305	0.185

*This table assumes that the Medium economic scenario holds*.

The results in Table 1 for the Medium demographic scenario align with those reported above for the overall, Medium scenario for the Australian generational economy. This is because combining the Medium demographic scenario with the Medium economic scenario yields the overall, Medium scenario.

The impact that fertility has on these measures of material living standards and intergenerational inequality can be elucidated by comparing the Medium demographic scenario with the Low Fertility and High Fertility scenarios in Table 1. Increasing fertility from low to medium to high increases the population share of younger Australians and decreases that of older Australians. These population changes are associated with very small decreases in the consumption growth rate. Through these effects on population and consumption growth, increasing fertility decreases intergenerational inequality as measured by the IGI index, although it appears to increase the indicator of equalizing redistribution to a small extent.

### Mortality

How mortality shapes material living standards and intergenerational inequality can be discerned by comparing the Medium demographic scenario with the Low Mortality and High Mortality scenarios in Table 1. Increasing mortality, which shortens life expectancies at birth, increases the population shares of younger Australians and Australians of middling age and decreases the population share of older Australians. These population changes are associated with increases in the consumption growth rate—at the cost of shorter life expectancies, of course. Through these effects on population and consumption growth, increasing mortality increases intergenerational inequality.

### Overseas Migration

The impact that overseas migration has on material living standards and intergenerational inequality can be discerned by comparing the Medium demographic scenario with the Low Migration and High Migration scenarios in Table 1. Increasing overseas migration from low to medium to high increases the population share of Australians of middling age and decreases that of older Australians, which is associated with increases in the consumption growth rate. Through these effects on population and consumption growth, increasing overseas migration decreases intergenerational inequality as measured by the IGI index, although it appears to increase the indicator of equalizing redistribution.

## Discussion

The results reported above describe how the Australian generational economy might perform in coming decades with respect to financial sustainability, intergenerational inequality, and material living standards. Four general points emerge from these results.

Firstly, because of population aging, age-specific, per capita consumption can only grow at a substantially lower rate than age-specific, per capita labor income if financial sustainability is to be maintained at a certain level. The difference between the labor-income growth rate and the consumption growth rate depends on the level at which financial sustainability is to be maintained and on the degree of population aging, and so on the demographic processes of fertility, mortality, and overseas migration. If the Medium demographic scenario holds in Australia between 2020 and 2120, and the time-interval support ratio is to be maintained at a level equal to the support ratio for 2020, generally speaking the consumption growth rate must be 0.23 percentage points below the labor-income growth rate.

Secondly, conflicts exist between the three evaluative criteria of financial sustainability, intergenerational inequality, and material living standards. Uncontrolled growth in material living standards places financial sustainability at risk. Even if growth in material living standards is controlled in such a way that financial sustainability is maintained at a certain level, growth in material living standards leads to intergenerational inequality. Resolution of this conflict between growth in material living standards and intergenerational inequality requires some degree of redistribution between birth cohorts.

In the overall, Medium scenario, the redistribution required is equal to 17.9% of the total present value of consumption between 2020 and 2120. Since in the Medium scenario cohorts born in later years enjoy higher levels of consumption than cohorts born in earlier years, this redistribution will involve redistribution from later, more-well-off cohorts to earlier, less-well-off cohorts. This redistribution can be instigated through a variety of mechanisms. In some cases the consumption of earlier, less-well-off cohorts can be raised through contemporaneous transfers from later to earlier cohorts. In some cases the consumption of earlier, less-well-off cohorts can be raised through these cohorts incurring liabilities that are subsequently repaid by later, more-well-off cohorts. There is also scope for the consumption of earlier cohorts to be raised through earlier cohorts drawing down their assets and transferring less to later cohorts at death through bequests. Older Australians tend not to draw down their assets as they age, with large amounts of assets being transferred to later cohorts at death (3, 30, 31). Needless to say, any decisions about redistribution from later to earlier cohorts should take into account the impacts of future demographic and economic changes as well as the uncertainties that surround the trajectories of these changes.

Thirdly, the conflict between growth in material living standards and intergenerational inequality can also be seen in the impacts that some demographic and economic changes have on these two evaluative criteria. Increasing the labor-income growth rate allows the consumption growth rate to be higher, but is also associated with rises in intergenerational inequality (as measured by the IGI index and the indicator of equalizing redistribution for consumption). Increasing fertility decreases intergenerational inequality (as measured by the IGI index), but is associated with very small decreases in the consumption growth rate. While increasing mortality is associated with increases in the consumption growth rate, this is at the cost of increases in intergenerational inequality, as well as shorter life expectancies. Notably, however, with regard to this conflict increasing overseas migration stands apart from these other demographic and economic changes. Increasing overseas migration is not only associated with increases in the consumption growth rate, it also decreases intergenerational inequality (as measured by the IGI index).

Fourthly, that increasing overseas migration combines increasing material living standards with decreasing intergenerational inequality suggests that it is a distinctly useful policy tool for meeting the challenges posed by population aging. This suggestion is strengthened by the fact that overseas migration is more amenable to direct state or government control than fertility, mortality, and labor-income growth. This is particularly true for countries like Australia that do not share land borders with any other countries.

The usefulness of increasing overseas migration as a policy tool suggests, in turn, that studies of financial sustainability, intergenerational inequality, and material living standards, both theoretical and empirical, should incorporate overseas migration where possible, in addition to fertility, mortality, and labor-income growth. This is particularly true for studies of countries like Australia in which overseas migration is significant and the total population size is comparatively small.

As described above, the assumptions that define the demographic and economic scenarios in this paper reflect levels or long-term trends observed over the last few decades. Nevertheless, it is possible that future demographic and economic changes will not reflect those experienced in the past. The COVID-19 pandemic has illustrated how expectations about future demographic and economic changes can be disrupted by unforeseen events (9, 10, 32). There is also a question about whether over extended time periods the labor-income and consumption growth rates discussed in this paper, when combined with population growth, are sustainable in environmental terms, irrespective of their sustainability in financial terms, particularly in the context of climate change. How technology will evolve in the future is also uncertain. The demographic and economic scenarios in this paper are based on a range of low, medium, and high assumptions for fertility, mortality, overseas migration, and labor-income growth, but the future trajectories of demographic and economic changes are inherently uncertain.

## Conclusion

This paper has examined how the Australian generational economy might perform in coming decades in terms of intergenerational inequality as well as financial sustainability and material living standards. How the performance of the Australian generational economy is shaped by variations in fertility, mortality, overseas migration, and labor-income growth has also been assessed.

In the future this work could be developed in a number of ways, including the delineation of scenarios that capture how the shape of the profile of per capita labor income by age in Australia might change in future years. The shape of this profile changed substantially during the 2000s as a result of increases in mature age labor force participation (3, 5), although change was much more moderate in the 2010s (33). The Australian government's 2021 Intergenerational Report includes projections of labor force participation rates that include some change in the age profile of participation rates in the future (9). Future change in the shape of the age profile of per capita labor income may affect the future performance of the Australian generational economy.

Evaluating the performance of a generational economy on the basis of intergenerational inequality, in addition to financial sustainability and material living standards, has the potential to foster a more complete understanding of that generational economy. It is hoped that this paper offers a step in this direction.

## Data Availability Statement

The datasets presented in this study can be found in online repositories. These repositories can be found at: https://cepar.edu.au/cepar-population-ageing-projections and https://www.ntaccounts.org/web/nta/show/Browse%20database.

## Author Contributions

Paper conceived and written by JR. Population projections provided by TW. Advice and ideas provided by JT and PM. Final manuscript reviewed by all authors. All authors contributed to the article and approved the submitted version.

## Funding

The Australian Research Council Centre of Excellence in Population Ageing Research (CEPAR) funds the salaries of Wilson and Temple and the estimation of the Australian National Transfer Accounts through Grant CE1101029. Additional funding has been received from the National Health and Medical Research Council and the Australian Research Council through an Ageing Well, Ageing Productively Research Program Grant (401158) and from the Australian Government Department of Social Services (46074597).

## Conflict of Interest

The authors declare that the research was conducted in the absence of any commercial or financial relationships that could be construed as a potential conflict of interest. The reviewer RL declared a past collaboration with one of the authors JR to the handling editor.

## Publisher's Note

All claims expressed in this article are solely those of the authors and do not necessarily represent those of their affiliated organizations, or those of the publisher, the editors and the reviewers. Any product that may be evaluated in this article, or claim that may be made by its manufacturer, is not guaranteed or endorsed by the publisher.
